# Breast Cancer Survivorship: the Role of Rehabilitation According to the International Classification of Functioning Disability and Health—a Scoping Review

**DOI:** 10.1007/s11912-022-01262-8

**Published:** 2022-04-11

**Authors:** Monica Pinto, Dario Calafiore, Maria Carmela Piccirillo, Massimo Costa, Ozden Ozyemisci Taskiran, Alessandro de Sire

**Affiliations:** 1 Rehabilitation Medicine Unit, Strategic Health Services Department, Istituto Nazionale Tumori -IRCCS - Fondazione G. Pascale, Naples, Italy; 2 Physical Medicine and Rehabilitation Unit, Department of Neurosciences, ASST Carlo Poma, Mantova, Italy; 3 Clinical Trials Unit, Translational Research Department, Istituto Nazionale Tumori - IRCCS – Fondazione G. Pascale, Naples, Italy; 4 Rehabilitation Medicine Unit, Department of Polyspecialistic Medicine, Cardarelli Hospital, Naples, Italy; 5 Department of Physical Medicine and Rehabilitation, Koç University School of Medicine, Istanbul, Turkey; 6 Department of Medical and Surgical Sciences, Physical Medicine and Rehabilitation, University of Catanzaro “Magna Graecia,” Catanzaro, Catanzaro, Italy

**Keywords:** Breast cancer 1, Survivorship 2, Rehabilitation 3, ICF 4, Health 5

## Abstract

**Purpose of Review:**

The population of breast cancer (BC) survivors is growing due to earlier diagnosis and effective combined treatments. A scoping review was performed to explore the role of rehabilitation in BC survivorship and the major issues in BC survivors with International Classification of Functioning Disability and Health (ICF) perspective.

**Recent Findings:**

The authors searched PubMed from January 1, 2018, up until November 9, 2021. The 65 selected publications were analyzed with the Comprehensive ICF BC Core Set (CCS) perspective and assigned to the categories of the CCS components along with the 3 areas of health (physical, mental, and social health). The multidimensional aspects of BC survivor disability are evident, whereas the topics of the articles concern several categories of the ICF BC CCS and all 3 areas of health. However, the current ICF BC CCS does not include certain categories related to emerging issues of BC survivorship recurring in the papers.

**Summary:**

Rehabilitation is crucial in BC survivorship management to give personalized answers to women beyond BC, and the ICF BC CCS remains an essential tool in rehabilitation assessment for BC survivors although it needs updating.

## Introduction

Breast cancer (BC) is the most frequent cancer in women worldwide, accounting for 11.7% of total cases, with 2.3 million cases newly diagnosed in 2020 [1•]. Due to earlier diagnoses and more effective treatment, the 5-year survival rate has continued to improve over the last two decades and is now 86% in Italy and Turkey [2]. Male BC cases are less than 1% of all diagnosed breast cancers [3, 4•]; therefore, where not specified, we refer to female BC patients.

With improving survival rates globally [5–7], many survivors experience short-term, long-term, and late effects from both the cancer and cancer-related treatments. These effects may result in mental, physical, and social health–related issues not only while in active treatment but also during long-term survivorship phase [8, 9]. In the last two decades, distinguished authors have addressed cancer survivorship [10, 11, 12•, 13–15] with both American Cancer Society and the American Society of Clinical Oncology publishing Breast Cancer Survivorship Care Guidelines [16] to support clinicians in the care for BC survivors. With growing population of BC survivors, the individual, family, and societal challenges for women beyond BC are becoming a critical issue in public health system and require an in-depth reorganization of survivorship care at the local, regional, and national levels. Survivorship care is a significant challenge for the future of BC and all cancers. Rehabilitation medicine is the most appropriate medical specialty for treating cancer survivors suffering from disability related to cancer itself and long-term side effects of treatments [17•].

Rehabilitation can be thought of both as “a general health strategy” [18] and as “a set of interventions” focused on enabling persons at risk or with physical and/or mental health disabling conditions to achieve and maintain optimal functioning and to pursue the best health-related quality of life in their family and social context [18, 19].

According to the International Classification of Functioning Disability and Health (ICF) [20] adopted by the World Health Organization (WHO) in 2001, cancer and its treatments affect body structures and influence body functions, activities, and participation, as well as environmental factors. Specific Comprehensive and Brief ICF Core Sets were subsequently developed for BC to describe function and disability [21].

Therefore, the aim of this scoping review is to define the role of rehabilitation in the BC survivorship within the context of the current ICF BC Core Sets, increase awareness of the needs in BC survivors, and support stakeholders, health professionals and governments to provide optimal care as well as contain healthcare costs.

## Methods

The authors, with the assistance of the librarian of the National Cancer Institute in Naples, provided a review of indexed PubMed articles from January 1, 2018, to November 9, 2021, using the keywords “breast cancer,” “survivorship,” and “rehabilitation.” Exclusion criteria were articles not in English (both text and abstract); published prior to 2018; including pharmaceutical interventions for cancer and/or treatment-related conditions (e.g., osteoporosis); including population without cancer (excluding non-cancer population as control group); nonhuman studies; letters to the editor, protocols, or preliminary results of major ongoing studies; including survivors with various cancer diagnoses together with BC, unless the results reported by specific cancer types; sample size less than 50 BC patients; specific for a subgroup of BC patients as such as BC and type 2 diabetes; and considering survivorship even during the active treatment phase and/or time after active treatment less than 1 year. Secondly, two of the authors hand-searched PubMed, based on their experience, for additional relevant literature about survivorship care although unrelated to rehabilitation, including the guidelines for BC survivorship and BC Survivorship Care Plans (BC SCPs).

Two reviewers independently extracted data from included studies using a customized data extraction table in Microsoft Excel. In case of disagreement, consensus was achieved by the decision of another reviewer. The following data were extracted: (1) first author, (2) publication year, (3) journal, (4) nationality, (5) type of study, and (6) patient-reported outcome measures.

**Fig. 1:**
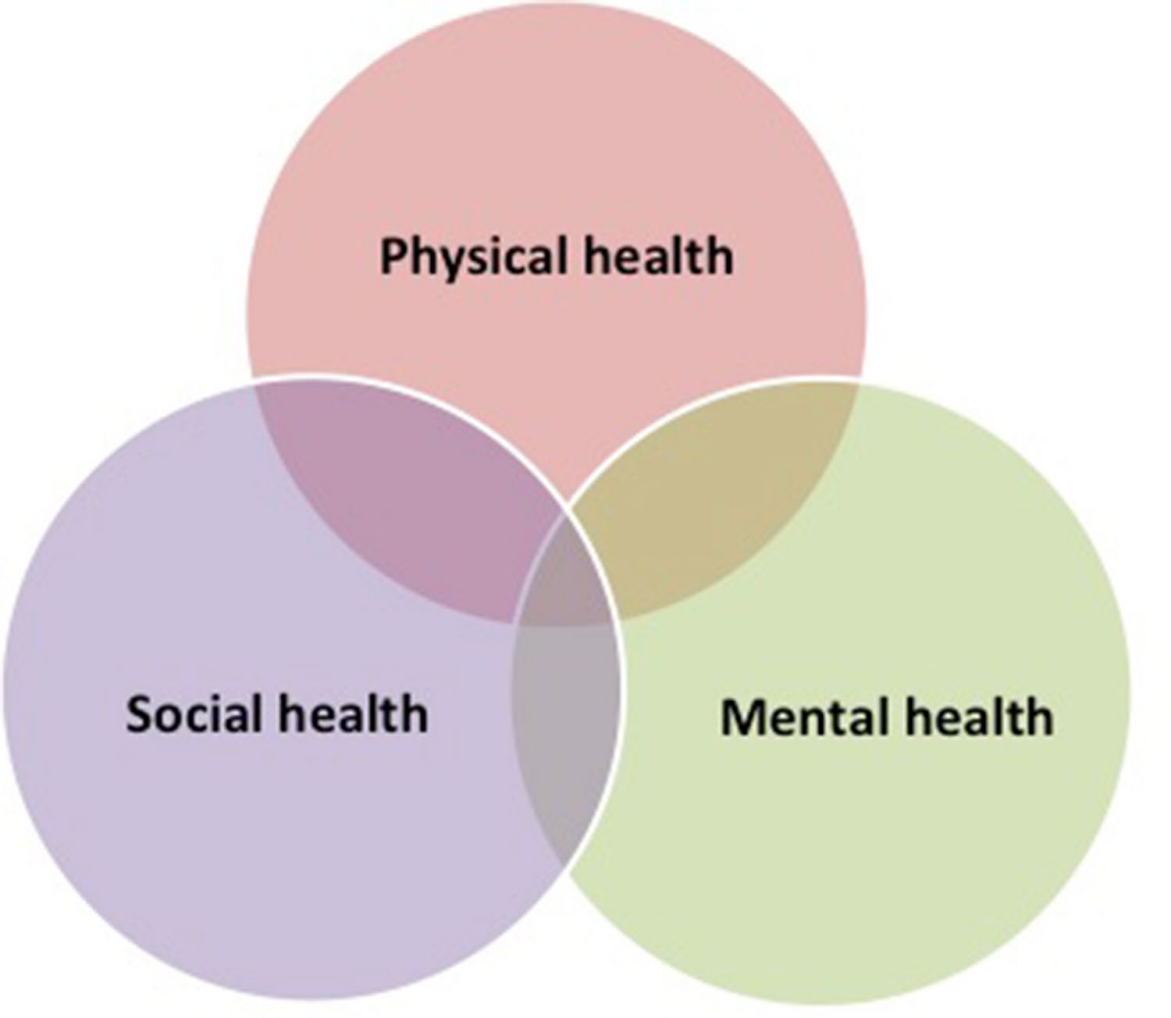
This figure describes the three areas of health (physical, mental, and social health) that are partly intersected

All the included publications were analyzed with the Comprehensive ICF BC Core Set (CCS) perspective, and were considered related to the Body Structure category, 3rd level s6302 breast and nipple. Therefore, considering the main topics of each paper, the selected articles were assigned to one or more of the ICF BC CCS major components (body function, activity and participation, environmental factors) and linked to specific categories. Finally, the same selected papers were assigned to one or more of the 3 areas of Health: Physical Health, Mental Health and Social Health (Fig. 1**)**.

## Results

A total of 181 publications were searched on PubMed. Following the exclusion criteria in the “Methods” section, 63 articles were selected and 118 were excluded. An additional 2 articles were selected by hand-searching; ultimately, 65 papers [22–30, 31••, 32–37, 38••, 39, 40, 41•, 42–61, 62•, 63–66, 67•, 68–70, 71•, 72•, 73–75, 76•, 77, 78•, 79–83, 84•, 85, 86] in total have been reviewed to extract the main topics and survivors needs (Table 1).

**Table 1:** Main characteristics of the publications included in the present review

Article	Journal	Country	Study design	Survivors needs
Chapman J et al. [22] 2018	British Journal of Health Psychology	Australia	RCT	Leisure activity, health-related quality of life
Bail JR et al. [23] 2018	Cancer	USA	RCT	Health-related quality of life, physical performance
Fong AJ et al. [24] 2018	Current Oncology	Canada	Cross sectional	Information about physical activity
Ghizzani A et al. [25] 2018	Gynecological Endocrinology	Italy	Review	Sexual activity
Gonzalez-Hernandez E et al. [26] 2018	Integrative Cancer Therapies	Spain	RCT	Quality of life, psychological status
Eaglehouse YL et al. [27] 2018	Journal of Cancer Survivorship	USA	Cross sectional	Health care services, insurance
Dieli-Conwright CM et al. [28] 2018	Journal of Clinical Oncology	USA	RCT	Metabolic syndrome, anthropometric variables, physical fitness
Igwebuike LT et al. [29] 2018	Supportive Care in Cancer	USA	Cross sectional	Community/home-based exercise
Mirandola D et al. [30] 2018	Supportive Care in Cancer	Italy	Cohort study	Health related quality of life, pain, fitness tests, mobility of shoulder-arm and spine flexibility
Stout NL et al. [31••] 2018	Journal of the National Cancer Institute	USA	Review	Physical rehabilitation
Dean LT et al. [32] 2018	Journal of Cancer Survivorship	USA	Cross sectional	Economic burden, mental and physical activity, perceived stress
Lee H et al. [33] 2018	Journal of Medical System	South Korea	Interview	User satisfaction
Cheng KKF et al. [34] 2018	Psycho-Oncology	Singapore	Cross sectional	Physical and psychological issues, patient care and support, health care system and information
Sabiston C et al. [35] 2018	Psycho-Oncology	Canada	Cross sectional	Physical activity, sedentary behavior, depression
Pullen T et al. [36] 2018	Psycho-Oncology	Canada	Cohort study	User satisfaction
Tsai E et al. [37] 2018	Psycho‐Oncology	USA	Qualitative study	Self-regulation physical activity
Cheville AL et al. [38••] 2019	American Journal of Physical Medicine and Rehabilitation	USA	Review	Integrated rehabilitative programs
Caperchione CM et al. [39] 2019	BMC Cancer	Australia	Cohort study	Physical activity, physical activity motivation, health-related quality of life
Singleton A et al. [40] 2019	BMC Cancer	Australia	RCT protocol	Text messages, self-efficacy for lifestyle outcomes, mood, health-related quality of life
Sheppard DM et al. [41•] 2019	BMJ Cancer	Australia	RCT protocol	Return to work, health related quality of life, occupational rehabilitation, support at work
Suzuki H et al. [42] 2019	Breast Cancer	Japan	Cohort study	Psychological distress, fatigue, self-efficacy
Sleight AL et al. [43] 2019	Disability and Rehabilitation	USA	Cross sectional	Support need care, health related quality of life
Schmidt ME et al. [44] 2019	European Journal of Cancer Care	Germany	Cross sectional	Return to work, global health‐related quality of life, physical, social, emotional, cognitive, role function, fatigue, arm morbidity, financial problems
Springfield S et al. [45] 2019	Journal of Cancer Survivorship	USA	Cross sectional	Physical activity, food frequency,
Tamminga SJ et al. [46] 2019	Journal of Cancer Survivorship	Netherlands	Cross sectional	Adverse work outcome, health related quality of life
Hamond R et al. [47] 2019	Journal of Cardiology	Japan	Cohort study	Cardiovascular diseases, job employment
Sabiston C et al. [48] 2019	Journal of Translational Medicine	Canada	Cohort study	Physical activity level
Arem H et al. [49] 2019	Journal of Alternative and Complementary Medicine	USA	Case–control study	Sleep disturbances
Ellegaard MB et al. [50] 2019	Journal of Cancer Education	Denmark	RCT protocol	Self management, patient education, empowerment
Cheng ASK et al. [51] 2019	Journal of Cancer Survivorship	China	Validity study	Anxiety and depression symptoms, work-related cognitive limitations, pain, fatigue
Lovelace et al. [52] 2019	Journal of Midwifery and Women’s Health	USA	Review	Psychological status, physical exercise, lifestyle habits
Hamood et al. [53] 2019	Journal of Occupational Rehabilitation	Israel	Cross sectional	Economic burden, return to work, health-related quality of life
Wolvers MDJ et al. [54] 2019	Journal of Occupational Rehabilitation	Netherlands	Cross sectional	Fatigue, return at work
Jalambadani Z et al. [55] 2019	Asia–Pacific Journal of Oncology Nursing	Iran	Case–control study	Health related quality of life
Coletta A et al. [56] 2019	PLOS ONE	USA	Retrospective study	Physical activity
Von Hippel et al. [57] 2019	Psycho-Oncology	USA	Cohort study	Sexual functioning
Myers JS et al. [58] 2019	Supportive Care in Cancer	USA	RCT	Cognitive impairment, Qigong, mindfulness-based exercise
Smith SK et al. [59] 2019	Supportive Care in Cancer	USA	RCT	Pain, depression, fatigue,
Lozano-Lozano M et al. [60] 2019	Annals of Physical and Rehabilitation Medicine	Spain	RCT	Health related quality of life, upper limb mobility, body composition
Czamanski-Cohen J et al. [61] 2020	BMJ open	Israel	RCT protocol	Depression, fatigue, pain, emotional awareness and expression
Cheng ASK et al. [62•] 2020	BMJ open	Hong Kong	RCT protocol	Problem solving, self-efficacy management, readiness for change, health-related quality of life
Tollosa DN et al. [63] 2020	Breast Cancer Research and Treatment	Australia	Longitudinal study	Self-reported physical activity, health behavior
Krok-Schoen JL [64] 2020	Cancer Control	USA	Pilot study	Survivorship care plan
Shih IH et al. [65] 2020	European Journal of Oncology Nursing	Taiwan	Cross sectional	Pain, sleep disturbances, fatigue, sexuality difficulties, depression, fear of recurrence
Invernizzi M et al. [66] 2020	Forntiers in Oncology	Italy	Review	Quality of life, risk assessment, new strategies
Montagnese C et al. [67•] 2020	Nutrients	Italy	RCT	Healthy life style, physical activity
Scott et al. [68] 2020	Psycho-Oncology	USA	Case–control	Memory, quality of life
Schmidt et al. [69] 2020	Patient Education and Counseling	Switzerland	Cross sectional	self-management support needs
Palmer NR et al. [70] 2020	Patient Education and Counseling	USA	Cross sectional	Fatigue, sexual problems, memory problems, occupational, emotional status
Davies C et al. [71•] 2020	Physical Therapy	USA	Clinical Practice Guideline	Lymphedema
Harrington SE et al. [72•] 2020	Physical Therapy	USA	Review	Physical performance status, health related quality of life, sexual function, fatigue
Krok-Schoen et al. [73] 2020	Supportive Care in Cancer	USA	Content analysis	Survivorship care plan
Matsuoka et al. [74] 2020	Journal of Cancer Survivorship	Japan	Interview	clinical issues including coordination between clinicians, life style, pain, work
Choi et al. [75] 2020	Breast Cancer Research and Treatment	USA	Content analysis	Survivorship care plan
Cheville et al. [76•] 2020	Journal of Cancer Survivorship	USA	Retrospective study	Health care utilization
Comander et al. [77] 2021	American Journal of Lifestyle Medicine	USA	Review	Physical activity, nutrition, sleep, social connection, psychological stress
Algeo N et al. [78•] 2021	BMC Cancer	Ireland	Systematic review	Occupational status
Mohlin A et al. [79] 2021	Current Oncology	Sweden	Narrative study	Pathographies and narrative of survivorship
Lewis J et al. [80] 2021	Disability and Rehabilitation	Australia	Scoping review	Cognitive difficulties at work, performance and participation impact of cognitive changes at work
Cha L et al. [81] 2021	Journal of Cancer Education	USA	Retrospective study	Fatigue, physical activity, mood, cognitive abilities, sexual health, sleep, pain, occupational status
Vega NJ et al. [82] 2021	Journal of Cancer Survivorship	USA	RCT	Cognitive function, mood status
Hiltrop K et al. [83] 2021	Psycho-Oncology	Germany	Cross sectional	Work, leisure and family status
Dorè I et al. [84•] 2021	Supportive Care in Cancer	Canada	Longitudinal study	Lifestyle behavior, depressive symptom, pain, fatigue
Sohn et al. [85] 2021	Supportive Care in Cancer	South Korea	Scoping review	Occupational status
Ryans et al. [86] 2021	Journal of Cancer Survivorship	USA	Clinical Practice Guidelines	Lymphedema

### Included Publications Per Country Per Year

Sixteen [22–30, 31••, 32–37] were published in 2018, twenty-three [38••, 39, 40, 41•, 42–60] in 2019, sixteen in 2020 [61, 62•, 63–66, 67•, 68–70, 71•, 72•, 73–75, 76•], and ten in 2021 [77, 78•, 79–83, 84•, 85, 86]. Twenty-eight studies were conducted in the USA [23, 27–29, 31••, 32, 37, 38••, 43–45, 49, 52, 56–59, 64, 68, 70, 71•, 72•, 73, 75, 76•, 77, 81, 82, 86], six in Australia [22, 39, 40, 41•, 63, 80], five in Canada [24, 35, 36, 48, 84•], four in Italy [25, 30, 66, 67•], three in Japan [42, 47, 74], two in Spain [26–30, 31••, 32–37, 38••, 39, 40, 41•, 42–60], two in South Korea [33, 85], two in Germany [44, 83], two in the Netherlands [46, 54], two in Israel [53, 61], one in Singapore [34], one in Denmark [50], one in China [51], one in Iran [55], one in Hong Kong [62•], one in Taiwan [65], one in Switzerland [69], one in Ireland [78•], and lastly one in Sweden [79].

### Design of the Included Studies

There were sixteen cross-sectional studies [24, 27, 29, 32, 34, 35, 43–46, 53, 54, 65, 69, 70, 83], nine randomized control trials (RCT) [22, 23, 26, 28, 58–60, 67•, 82], nine reviews [25, 31••, 38••, 52, 66, 71•, 72•, 77, 86], nine cohort studies [30, 36, 39, 42, 48, 57], five RCT protocols [40, 41•, 50, 61, 62•], three case–control studies [49, 55, 68], two interviews [33, 74], two retrospective studies [56, 81], two longitudinal studies [63, 84•], two content analysis [73, 75], two scoping reviews [80, 85], one qualitative study [37], one validity study [51], one pilot study [64], one systematic review [78•], and finally one narrative study [79] (see Table 1 for further details).

### Topics of the Included Publications According to Comprehensive ICF BC Core Set

Table 2 depicts second-level categories of the ICF BC CCS and highlights those related to the topics of the selected publications.

**Table 2:** Comprehensive ICF Breast Cancer Core Set categories (categories linked to the topics of the selected papers in bold characters)

Categories of the component Body functions	Categories of the component Activities and participation	Categories of the component Environmental factors
**b126 Temperament and personality functions b130 Energy and drive functions b134 Sleep functions b152 Emotional functions b180 Experience of self and time functions** b265 Touch function **b280 Sensation of pain b435 Immunological system functions b455 Exercise tolerance functions b530 Weight maintenance functions b640 Sexual functions b650 Menstruation functions** b660 Procreation functions b670 Sensations associated with genital and reproductive functions **b710 Mobility of joint functions** b720 Mobility of bone functions **b730 Muscle power functions b740 Muscle endurance functions b780 Sensations related to muscles and movement functions** b810 Protective functions of the skin b820 Repair functions of the skin b840 Sensation related to the skin	**d177 Making decisions d230 Carrying out daily routine d240 Handling stress and other psychological demands** d430 Lifting and carrying objects **d445 Hand and arm use** d510 Washing oneself d520 Caring for body parts d530 Toileting d540 Dressing **d550 Eating** d560 Drinking d570 Looking after one’s health **d620 Acquisition of goods and services d630 Preparing meals d640 Doing housework** d650 Caring for household objects d660 Assisting others d720 Complex interpersonal interactionsd750 Informal social relationships d760 Family relationships **d770 Intimate relationships d850 Remunerative employment d920 Recreation and leisure**	**e110 Products or substances for personal consumption** e115 Products and technology for personal use in daily living 165 Assets e225 Climate e310 Immediate family e315 Extended family e320 Friends e325 Acquaintances, peers, colleagues, neighbors and community members e340 Personal care providers and personal assistants **e355 Health professionals** e410 Individual attitudes of immediate family members e415 Individual attitudes of extended family members e420 Individual attitudes of friends **e425 Individual attitudes of acquaintances, peers, colleagues, neighbors and community members** e440 Individual attitudes of personal care providers and personal assistants e450 Individual attitudes of health professionals e465 Social norms, practices and ideologies e540 Transportation services, systems and policies e555 Associations and organizational services, systems and policies **e570 Social security services, systems and policies** e575 General social support services, systems and policies **e580 Health services, systems and policies e590 Labor and employment services, systems and policies**

Among the component Body functions, the most explored categories in the papers, in order of frequency, are b455 Exercise tolerance functions (28 papers), b730 Muscle power functions (16 papers), b740 Muscle endurance functions (15 papers), b530 Weight maintenance functions (14 papers), b435 Immunological system functions including b435.2 and b435.3 related to lymphedema (9 papers), b640 Sexual functions (6 papers), and b650 Menstruation functions (2 papers).

Among the component Activities and participation, the most explored categories, in order of frequency, are d240 Handling stress and other psychological demands (18 papers), d230 Carrying out daily routine (11 papers), d850 Remunerative employment (8 papers), d920 Recreation and leisure (5 papers), d445 Hand and arm use (4 papers); d550 Eating (2 papers), d177 Making decisions (2 papers), d770 Intimate relationships (2 papers), d620 Acquisition of goods and services (1 paper), d630 Preparing meals (1 paper), and d640 Doing housework (1 paper).

Moreover, among the component Environmental factors, the most explored categories are e580 Health services, systems and policies (32 papers); e590 Labor and employment services, systems, and policies (10 papers); e355 Health professionals (8 papers); e570 Social security services, systems, and policies (6 papers); e110 Products or substances for personal consumption (1 paper); e450 Individual attitudes of health professionals (1 paper); and e425 Individual attitudes of acquaintances, peers, colleagues, neighbors, and community members (1 paper).

Considering the three areas of health, 8 articles cover all the 3 areas of health, another 8 cover both physical and mental health, 1 covers both physical and social health, 2 cover both mental and social health, 9 cover physical health, 7 cover mental health, and 15 cover social health. The authors also considered 15 papers that are not matched with any area of health but concern health system and policy.

## Discussion

Given the presented studies, it is clear that rehabilitation is an essential part of cancer care in all phases of disease, and especially in survivorship, when the priority of interventions is focused on persons who experienced cancer and need to return to their family and social life. This is a common goal across all types of cancer, and there are many high-quality studies on assessing survivorship in a mixed populations of a variety of cancer diagnoses. However, there is no international consensus on the time framework of survivorship care.

We focused our attention on early or long-term survivorship at least 1 year after the end of active treatments considering the rehabilitation strategy prevalent in the posttreatment phase better than in the acute phase when cancer curative strategy is the main goal [87]. To guarantee the continuum of care beyond diagnosis and active treatment until survivorship, the Survivorship Care Plan (SCP) model was developed. In several countries, oncologists have adopted this model involving general practitioners (GPs) and primary care physicians (PCPs) in the survivorship care. The SCP provides information on cancer and related treatments along with instructions for follow up care; however, equally detailed post-treatment side effects are less frequently included. Despite the recommendations from major bodies and scientific societies (American Cancer Society, American College of Surgeon, and others) to implement the SCPs, there is no strong evidence that SCPs positively impact on level of outcomes in cancer survivors [88•, 89•]. Specific BC SCPs are available including a brief clinical summary and the follow-up planning rather than recommendations to support survivors and their family in carrying out daily life, adopting healthy behaviors, and following rehabilitation programs if indicated [75]. This strategy is still far from optimal. GPs and PCPs have limited time and resources to provide appropriate interventions for a cancer survivor’s physical, mental, and social health needs, including education and empowerment, while continuing to provide comprehensive medical care. However, the addition of the involvement of a physiatrist’s focus on rehabilitation needs and function could specifically address a survivor’s physical, mental, and social health needs.

The multidimensional aspect of BC-related disability is evident considering the assignment of the selected papers to the major components of ICF BC CCS and included categories based on the topics of each paper. A paper is commonly assigned to a couple or more categories of the ICF BC CCS major components but less frequently to 2 or 3 of the areas of health. Out of the 65 selected articles, 31 papers cover a single area of health and 15 are not matched with any area of health because they concern health system and policy.

However, many categories of the ICF BC CCS are not linked to the items of selected papers. On the other hand, the ICF BC CCS does not include many categories recurring in the analyzed papers. In our opinion, the ICF BC Comprehensive and Brief Core Sets are a valuable tool, but they need to be updated. Significant categories are lacking in the BC CCS such as b144 Memory function, b164 High level cognitive function, e125 Products and technology for communication, e130 Products and technology for education, e135 Products and technology for employment, and e140 Products and technology for culture, recreation, and sports. Memory loss and cognitive decline are frequent complaints from BC survivors and can strongly impact their return to work and the maintenance of the same level of pay.

There is a growing interest in the use of computer technology and social media to support BC survivors by providing resources and information regarding physical activity, healthy behavior, cognitive enhancement, and vocational training. Financial burden and loss of work opportunity are strategic issues for individuals and families as well as for society. There are notable disparities in survivors returning to work and sustaining financial burden between developed and underdeveloped countries as well as various socioeconomic groups and geographic (rural versus urban) areas of the same country. Items included between “body functions” and “activity and participation” are traceable to the areas of physical and mental health. Among these problems, some issues have been very well represented for decades such as b435 Lymphedema, fatigue; b455 Exercise tolerance functions; and b740 Muscle endurance functions, psychological needs (d240 Handling stress and other psychological demands). However, new needs are emerging including weight management, sexual and intimate life difficulties, and daily challenges that have been less reported in previous rehabilitation studies. These issues are evident in many studies on quality of life (QoL) which is a significant outcome in cancer care and a substantial endpoint in many clinical trials. QoL is a powerful indicator for outcomes in all chronic diseases as well as in cancer. Among all QoL measurement scales, the most used remains the MOS 36-item Short-Form Health Survey (SF-36) that is widely validated in several chronic diseases. However, SF-36 is not cancer specific [90]. There are current studies to develop specific cancer survivorship QoL measurement instrument [91•]. In our opinion, it will be successful if QoL measurement becomes an essential step in survivor’s rehabilitation assessment to further highlight the role of rehabilitation in cancer survivorship care. As social health is a key factor to QoL, it will be important to leverage technology to help provide information and support for the self-management of a healthy lifestyle including nutrition and physical activity.

BC survivors are asking for support from the health care systems and policies; however, these systems are still far from offering satisfactory support to patients and families. The reality of cancer care is changing with increasing prevalence of BC survivors, needing interventions covering aspects of physical, mental, and social health even after their active treatment and surveillance have completed. Rehabilitation services could be the bridge between the comprehensive cancer center–based model and primary care–based model, offering each cancer survivor a tailored treatment along with an individual rehabilitative plan including and not excluding the model of SCP. This paper is far from giving a certain answer on how to manage cancer survivorship, but we hope to identify the need for supporting policy and health system changes to bridge the gap between active treatment and survivorship beyond cancer.

## Conclusions

The findings of this scoping review report that BC survivors are a growing population with emerging issues that strongly impact their life. The ICF BC CCS, along with its brief version, remains an essential tool in rehabilitation assessment for BC survivors even though it needs updating and to be associated with quality of life evaluation scales. The role of rehabilitation is crucial in BC survivorship both to give personalized answers to women beyond BC and to support the proper allocation of available resources for survivorship in each country within their means.

## Data Availability

Data and material are available at https://zenodo.org/record/6372904#.Yj61FXMLIU acceded on 21/03/2022.
